# Effect of PKM2 on *M. tuberculosis* Rv1987-induced macrophage M2 polarization

**DOI:** 10.3389/fcimb.2026.1740892

**Published:** 2026-02-13

**Authors:** Wenzhen Wang, Hanyu Yang, Guoying Deng, Yufang Ma, Shanshan Sha

**Affiliations:** 1 Department of Biochemistry and Molecular Biology, College of Basic Medical Sciences, Dalian Medical University, Dalian, China; 2 The Second Hospital of Dalian Medical University, Dalian Medical University, Dalian, China; 3 The Queen’s University of Belfast Joint College, China Medical University, Shenyang, Liaoning, China; 4 Department of Microbiology, College of Basic Medical Sciences, Dalian Medical University, Dalian, China

**Keywords:** M2 polarization, macrophage, *Mycobacterium tuberculosis*, PKM2, tuberculosis

## Abstract

**Introduction:**

Mycobacteria induce host macrophage M2 polarization to construct a kindly environment for their intracellular growth. In our previous study, we found that *M. tuberculosis* Rv1987 protein induced macrophage polarization to M2-like phenotype. However, little is known about the changes of host metabolites and the effects of related enzymes in this process.

**Methods:**

Here, using our previously constructed infection model by *M. smegmatis* overexpressing Rv1987 protein, we analyzed the alterations of energy metabolism-related metabolites and the function of M2 isoform of pyruvate kinase (PKM2), the key enzyme of glycolysis, in mycobacteria-induced M2 macrophages.

**Results:**

The results showed that the expression, enzyme activity and nucleus translocation of PKM2 were all impaired in Rv1987-induced M2 macrophages. Activation of PKM2 by its activator TEPP-46 reversed the M2 polarization and enhanced the inflammation of macrophages, and subsequently reduced the mycobacterial load in mouse lung tissues during infection.

**Conclusion:**

All these results suggested that host PKM2 is closely associated with *M. tuberculosis* Rv1987-induced M2 polarization, which can be considered as an intervention target in anti-tuberculosis therapy.

## Introduction

Tuberculosis (TB) remains the leading cause of death from a single infectious agent globally. In 2023, a total of 8.2 million people were reported as newly diagnosed with TB and 1.25 million people were dead from TB (World Health Organization, 2024). Compared with 2015, only 8.3% and 23% reduction in TB incidence rate and number of TB deaths (World Health Organization, 2024). More efforts including developing new drugs and vaccines are needed for ending global TB epidemic. Understanding *Mycobacterium tuberculosis* (Mtb)-host interactions especially the key molecules that medicate these processes will greatly contribute to drug and vaccine discoveries.

Macrophages are the first immune-response cells during Mtb infections. At the early stage of infection, alveolar macrophages polarize to classically activated phenotypes (M1), but finally differentiate to alternatively activated phenotypes (M2) that permits the growth of Mtb (Khan et al., 2019). Compared with M1 macrophages, M2 macrophages exhibit anti-inflammatory, low antigen presentation, and low production of inflammatory cytokines, thus provide a friendly environment for TB development especially in MDR-TB/XDR-TB cases (Khan et al., 2019; Cho et al., 2020). However, very little is known about the mechanism how Mtb induces alveolar macrophage into M2 macrophage so far, including the key bacterial components that may be potential drug targets, and the crucial host enzymes and signal molecules involved in M2 polarization.

Rv1987 is a secretory protein encoded by the *rv1987* gene present in the region of difference-2 (RD2) of the Mtb genome, which is closely associated with Mtb virulence (Parkash et al., 2009; De Souza et al., 2011). In our previous study, we found that Rv1987 stimulated macrophage toward M2 polarization through activating PI3K/Akt1/mTOR signal pathway (Sha et al., 2021). In that study, some metabolites and cellular metabolism-related enzymes were found significantly changed in Rv1987-induced M2 macrophages. The lactate level significantly decreased compared with the control group. Meanwhile, the expressions of arginase-1 (Arg1) that is related to arginine metabolism, COX2 that is involved in oxidative phosphorylation (OXPHOS), and Pfkfb3 that is responsible for glycolysis, were also dramatically altered in Rv1987-induced M2 macrophages. All these results suggested that cellular metabolism plays an important role in the M2 polarization of macrophages induced by mycobacteria, but its extract mechanism remains unclear. In recent years, using LPS/IFN-γ and IL-4-induced models, macrophage polarization was found to be accompanied by different metabolic pathways, including glycolysis, tricarboxylic acid (TCA) cycle, fatty acid oxidation, and the pentose phosphate pathway (PPP) (Van den Bossche et al., 2017). LPS-induced M1 macrophages always have an enhanced glycolysis but a disrupted TCA cycle to meet the requirement for their pro-inflammation response, while IL-4 induced M2 macrophages are charactered with a fluent TCA cycle, OXPHOS and fatty acid oxidation to support their anti-inflammatory functions (Van den Bossche et al., 2017). IFN-γ- programmed M1 macrophages have an increased expression of innate immunity regulatory genes (Inregs), while IL-4-programmed M2 macrophages exhibit reduced Inregs expression (Khan et al., 2022). Multi-OMICs analysis on the peripheral blood mononuclear cell (PBMC)-differentiated macrophages reveals that IFN-γ-induced M1 macrophages have reduced histone acetylation levels but high levels of acetylated amino acids, while IL-4-induced M2 macrophages exhibit high levels of immune-suppressive effectors kynurenine and serotonin (Sowers et al., 2022). These changes are not only essential for the different energy requirement and material biosynthesis of macrophage polarization, but also facilitate the clearance or growth of Mtb in macrophage (Huang et al., 2019).

Some enzymes are reported to be crucial for macrophage polarization. For example, glycolytic enzyme 6-phosphofructo-2-kinase/fructose-2, 6-biphosphatase 3 (PFKFB3) (Chen et al., 2022), glyceraldehyde 3-phosphate dehydrogenase (GAPDH) (Millet et al., 2016), and pyruvate kinase (Weng et al., 2023) are found to be upregulated M1 macrophages and closes associated with their inflammatory functions. In contrast, TCA cycle related enzymes, such as pyruvate dehydrogenase (PDH) (Palmieri et al., 2023), isocitrate dehydrogenase (IDH1) (Jha et al., 2015) and succinate dehydrogenase (SDH) (Lampropoulou et al., 2016), are suppressed in M1 macrophages, which leading the accumulation of intermediate products in mitochondria. Compared to M1, the situation in M2 cells seems calmer with a normal or slightly enhanced glycolytic and TCA enzymes. Among these enzymes, pyruvate kinase is one of the most studied especially in cancer development (Israelsen and Vander Heiden, 2015). It catalyzes the conversion of phosphoenolpyruvate and ADP to pyruvate and ATP in glycolysis and plays a role in regulating cell metabolism. There are four pyruvate kinase isoforms and the M2 isoform of pyruvate kinase (PKM2) is expressed both in cancers and normal tissues and regulates cell metabolism in a more flexible approach. Different from other isoforms of pyruvate kinase that exist in stable tetramers, PKM2 has the form of tetramers, dimers, or even monomers (Israelsen and Vander Heiden, 2015). The tetramer form is essential for PKM2 enzymatic activity (Ashizawa et al., 1991), while the succinylated dimer form of PKM2 can translocate to nucleus and enhance the expression of IL-1β (Li et al., 2025). In macrophages, PKM2-mediated glycolysis promotes inflammasome activation by modulating EIF2AK2 phosphorylation in macrophages (Xie et al., 2016). In LPS-induced M1 macrophages, PKM1 expression has no change but PKM2 is significantly upregulated. The LPS-induced dimeric/monomeric PKM2 enters into a complex with Hif-1α, which can directly bind to the IL-1β promoter to enhanced the production of IL-1β (Palsson-McDermott et al., 2015). It was also reported that nuclear dimer PKM2 functions as a protein kinase that phosphorylates the transcription factor STAT3, thus boosting IL-6 and IL-1β production (Shirai et al., 2016). The activation of PKM2 by small molecule TEPP-46 promotes PKM2 tetramer formation but inhibits the translocation of PKM2 dimer into nucleus, thus totally attenuates the IL-1β production and induces macrophage polarization to M2 phenotype (Palsson-McDermott et al., 2015).

However, above studies are mostly performed in the cytokine or LPS-induced macrophages, which are the extremes of cellular metabolism. How Mtb regulates macrophage metabolism, especially the function of metabolic enzymes in this process, is still poorly understand. Actually, it is reported that stimulation of macrophages polarization with different stimuli showed distinct activation profiles including non-M1/M2 subsets (Xue et al., 2014). Therefore, it is essential to study the metabolic alterations in mycobacteria-infected macrophages, which will help us to clarify the specificity of bacterium-induced macrophage polarization and understand the different effect of eukaryotic enzymes on this process. Here, we profiled energy metabolism-related metabolites and studied the function of PKM2 in Mtb Rv1987-induced M2 polarization of macrophages. PKM2 activator TEPP-46 was used to reverse the M2 polarization *in vitro* and *in vivo* and its anti-mycobacteria effect was evaluated. The findings firstly clarified the function and regulation of host PKM2 in Mtb protein-induced M2 polarization, which provides a novel strategy for anti-tuberculosis therapy.

## Materials and methods

### Bacteria, cells and animals

The *M. smegmatis* mc^2^155 overexpressing Rv1987 protein (MS1987) and *M. smegmatis* containing empty vector (MSVec) were constructed previously (Sha et al., 2017) and cultured in LB broth containing 0.05% Tween 80 and 25 µg/mL kanamycin. Murine macrophage cell line Raw264.7 cells was obtained from ATCC and cultured in DMEM medium containing 10% fetal bovine serum, 100 U/mL penicillin and 100 µg/mL streptomycin. Male C57BL/6J mice (6–8 weeks old) were obtained from Liaoning Changsheng Biotech Company with permission number SCXK (Liao) 2020–0001 and maintained in clear environment.

### Infection of macrophages

RAW264.7 cells were cultured in 6-well (1×10^6^ cells per well) or 10 cm plates (1×10^7^ cells per plate) and infected with MS1987 or MSVec at an MOI of 20:1 for 3 hours. After washed by PBS for three times, the cells were cultured with fresh medium for additional 0, 4, 6, 16, or 24 hours. For TEPP-46 experiment, the cells were pre-treated with 30 or 50 µM TEPP-46 for 1 hour, then infected with bacteria. The cells were finally collected for RNA isolation, total protein preparation, cytoplasmic and nuclear protein preparation, or DSS treatment for PKM2 tetramer analysis.

### Detection of energy metabolism-related metabolites by liquid chromatography-mass spectrometry/MS

RAW264.7 cells at 24 hours post-infection were collected from 10 cm plates, washed by PBS, and quickly frozen in liquid nitrogen. Five independent experiments were set for each group. During the measurement, add 0.5 mL of methanol acetonitrile aqueous solution (2:2:1, v/v) to the sample (6.5×106 cells for each sample), vortex for 1 min, and ultrasound for 30 min on ice. The samples were paced at -20 °C for 1 hour to precipitate proteins, then centrifuged at 14000 ×g at 4 °C for 20 min. The supernatant was transferred to another tube and freeze-dried for LC-MS/MS analysis.

The samples were separated using an Agilent 1290 Infinity LC ultra-high performance liquid chromatography system (Agilent, German). Mobile phase included solution A (10 mM ammonium acetate aqueous solution) and solution B (acetonitrile). The liquid phase gradient was as follows: 0–18 min, liquid B changing linearly from 90% to 40%; 18-18.1 min, B solution changing linearly from 40% to 90%; 18.1–23 min, maintaining solution B at 90%. The column temperature was 45 °C and the flow rate was 300 µL/min. The peak area and retention time was analyzed by Multiquant software.

The metabolites were identified by mass spectrometry using a 5500 QTRAP mass spectrometer (AB SCIEX) in negative ion mode. The conditions are as follows: source temperature 450°C; ion source gas1(Gas1):45; ion source gas2(Gas2):45; curtain gas (CUR):30; ionSapary voltage floating (ISVF): 4500 V. The ion pairs were detected in MRM mode.

### Concentration determination of citrate and succinate

The citrate and succinate contents in macrophages were accurately quantified by high performance liquid chromatography (HPLC) with citrate and succinate standards. RAW264.7 cells at 24 hours post-infection were collected from 10 cm plates and quickly frozen in liquid nitrogen. During the measurement, add 1 mL of cold water per 0.1 g sample and placed at 4°C overnight. The sample was then centrifuged at 8000 ×g for 10 min at 4 °C and the supernatant was used to perform HPLC detection with Sepax C18 reverse phase chromatography column (250 mm * 4.6 mm, 5 µm) by Rigol L3000 HPLC machine. The mobile phase was 16.25 mM NaH_2_PO4 (pH 4-5) containing 2% methanol. A volume to 10 μL sample was applied and the flow rate was set at 0.8 mL/min. The column temperature is 30 °C, the deformation time is 40 min, and the UV detection wavelength is 214 nm.

### Detection of SDH and PKM2 activity

RAW264.7 cells at 6 hours post-infection were collected from 6-well plates and SDH and PKM2 enzyme activities were determined. SDH activity was detected using CheKine™ SDH Activity Assay Kit (Abbkine, Catalog no. KTB1230). PKM2 activity was detected by PK activity Assy Kit (MYCKLIN, China, Catalog no. P930578).

### Infection of mice

C57BL/6 mice were challenged with MS1987 or MSVec for 7 days at 5×10^9^ colony forming units (CFU)/day/mouse by inhalation as described previously (Sha et al., 2017; Liu et al., 2023). The bacteria were collected freshly according to the bacterial OD_600_-CFU standard curve, suspended in 4 mL PBS buffer, and finally aerosolized using aerosol generator (Kangjie Instrument, China). Five mice were assigned in one group. In TEPP-46 treatment group, the mice were pre-treated with TEPP-46 at the dose 10 mg/kg by intraperitoneal injection at 4 hours before infection with MS1987 bacteria. The mice were sacrificed by cervical dislocation without anesthesia at day 9 and 11 after the first day of infection, and the lung tissues were isolated sterilely for bacterial CFU counting and total RNA isolation, and the serum was collected for IL-1β determination.

### RNA isolation and qPCR

The RAW264.7 cells in 6-well plates or about 50 mg lung tissues were used for the isolation of total RNA with the RNAiso plus reagent (TaKaRa, Otsu, Japan) according to the manufacturer’s instructions. The concentration of RNA was determined through detecting its absorbance at 260 nm. After that, 1 µg of isolated RNA was immediately reverse transcribed to cDNA using the PrimeScriptTM RT reagent kit with gDNA Eraser (TaKaRa). The expression of PKM2, IL-1β, IL-12p40, iNOS, IL-10, Arg1, TNF-a was analyzed using RT-qPCR with the TB Green^®^ Premix Ex TaqTM II kit (TaKaRa). The primers are listed in **Supplementary Table 1**.

### Protein preparation

For total protein preparation, the RAW264.7 cells in 6-well plates were washed by PBS and hydrolyzed by 200 µL RIPA reagent containing 1 mM phenylmethyl sulfonylfluoride (PMSF). The lysate was placed on ice for 30 min and votexed for 30 s at intervals of 10 min. After centrifugation at 12000 × g for 10 min, the supernatant was collected for western blot analysis. For cytoplasmic and nuclear proteins preparation, the cells were treated with nucleus extraction reagent in Nucleus Proteins Isolation Kit (Solarbio, China, catalog no. R0050), placed on ice for 10 min, vortexed for 10 sec, and finally centrifuged at 12000 ×g for 10 min. The supernatant contained cytoplasmic proteins and the pellet contained nuclear proteins.

### Disuccinimidyl suberate treatment

For PKM2 tetramer analysis, the RAW264.7 cells were collected from 10 cm plates and added by 400 μL PBS containing 5 mM DSS. Incubate the mixture at room temperature for 60 min for protein crosslinking. Quench the reaction by adding 20 mM Tris-HCl and place the mixture at room temperature for 15 min. Collect the cells by centrifugation and then hydrolyzed by adding 300 µL RIPA reagent containing 1 mM PMSF to prepare the DSS-treated proteins.

### Western blot

The concentrations of total protein, cytoplasmic protein, nuclear protein, and DSS-treated protein samples were firstly quantified by BCA methods. An amount of 20 µg total protein, 50 µg cytoplasmic protein, or 50 µg nuclear protein, was used for western blot detection of the expression and isoform structure of PKM2.

### ELISA

The mouse serum was prepared by centrifugation of mouse blood at 1500 ×g for 10 min for 2 times. The levels of IL-1β and TNF-α in mouse serum were determined by ELISA kit (UpingBio technology Co. Ltd, China).

### CFU counting

For determination of bacterial load in mouse lung, 80 mg lung tissues were cut into small pieces and homogenized in 1 mL sterile PBS buffer. The homogenates were serially diluted with PBS and plated on LB agar plates containing 25 mg/mL kanamycin and incubated at 37 °C. The bacterial CFU was counted after 3 days.

### Lung histopathology

The lower lobe of right lung was obtained from the infected mice and fixed with 10% formalin in PBS. Pathological slides were prepared and stained using H&E as described in report (Basaraba et al., 2006). The slides were finally observed with CaseViewer 2.4 software (3DHISTECH Ltd. Budapest, Hungary).

### Statistical analysis

Statistical analyses were performed using GraphPad Prism 9 software. One-way ANOVA analysis with *post-hoc* Tukey’s multiple comparisons was used to determine the statistical significance between groups, defined as *P < 0.05, **P < 0.01, ***P < 0.001 and ****P < 0.0001.

## Results

### The energy metabolism was altered in Rv1987-induced M2 macrophages

**Figure 1:**
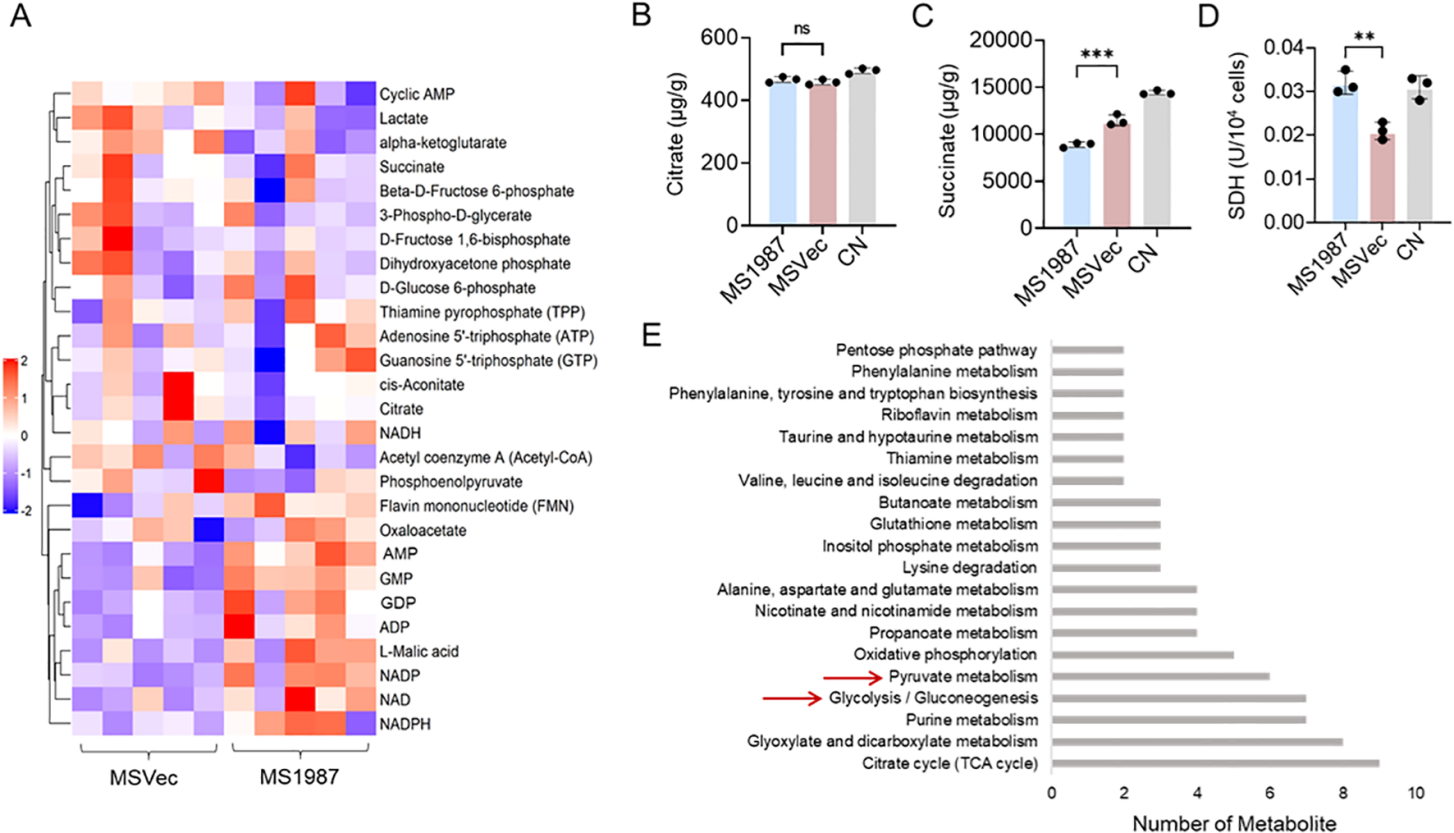
The energy metabolism was altered in Rv1987-induced M2-like macrophages. The RAW264.7 cells were infected by MS1987 or MSVec for 3 hours and collected for metabolites analysis at 24 hours post-infection. **(A)** Energy metabolome analysis by LC-MS/MS. Five repeats were set for each group. **(B, C)** Concentrations detection of succinate and citrate by HPLC. **(D)** SDH activity detection. **(E)** KEGG analysis of different metabolites. The data in **(B–D)** are shown as the mean ± SD (n = 3), pooled from three independent experiments. Mean values were compared using one-way ANOVA analysis with *post-hoc* Tukey’s multiple comparisons test (**P < 0.01; ***P < 0.001; ns, no significant difference). MS1987, *M. smegmatis* overexpressing Mtb Rv1987 protein; MSVec, *M. smegmatis* containing empty vector pVV2; CN, control group without infection.

To study the energy metabolism in Rv1987-induced M2-like macrophages, MS1987 overexpressing Rv1987 protein and the control strain MSVec containing empty vector were used to infect RAW264.7 macrophages. At 24 hours post-infection, RAW264.7 cells were collected and their energy generation-related metabolites were analyzed by LC-MS/MS. The results showed that the energy metabolism profiles were different in MS1987-infected macrophages compared to MSVec control group (**Figure 1A**). The significantly increased GMP, GDP, AMP and ADP and the decreased glycolysis intermediate products such as fructose 1, 6-bisphosphate and lactate indicated a slow generation of energy in MS1987-induced M2 macrophages. The less accumulation of succinate, malic acid, α-ketoglutarate suggested the enhanced flux through the TCA cycle in MS1987-infected macrophages. To confirm these results, the concentrations of citrate and succinate in MS1987- and MSVec-infected macrophages were further detected by HPLC and the consistent results were obtained (**Figures 1B, C**). Enzyme activity analysis found an elevated SDH activity in MS1987-infected macrophages compared to MSVec-infected cells (**Figure 1D**), which may be the reason why succinate was not accumulated in MS1987-infected cells. KEGG analysis showed that the different metabolites were more enriched in TCA cycle, glycolysis, purine metabolism and pyruvate metabolism (**Figure 1E**). All these results suggested that the energy metabolism play important roles in the process of Mtb-Rv1987 induced M2 polarization of macrophage.

### The expression, enzymatic activity and tetramerization of PKM2 were suppressed in Rv1987-induced M2 macrophages

**Figure 2:**
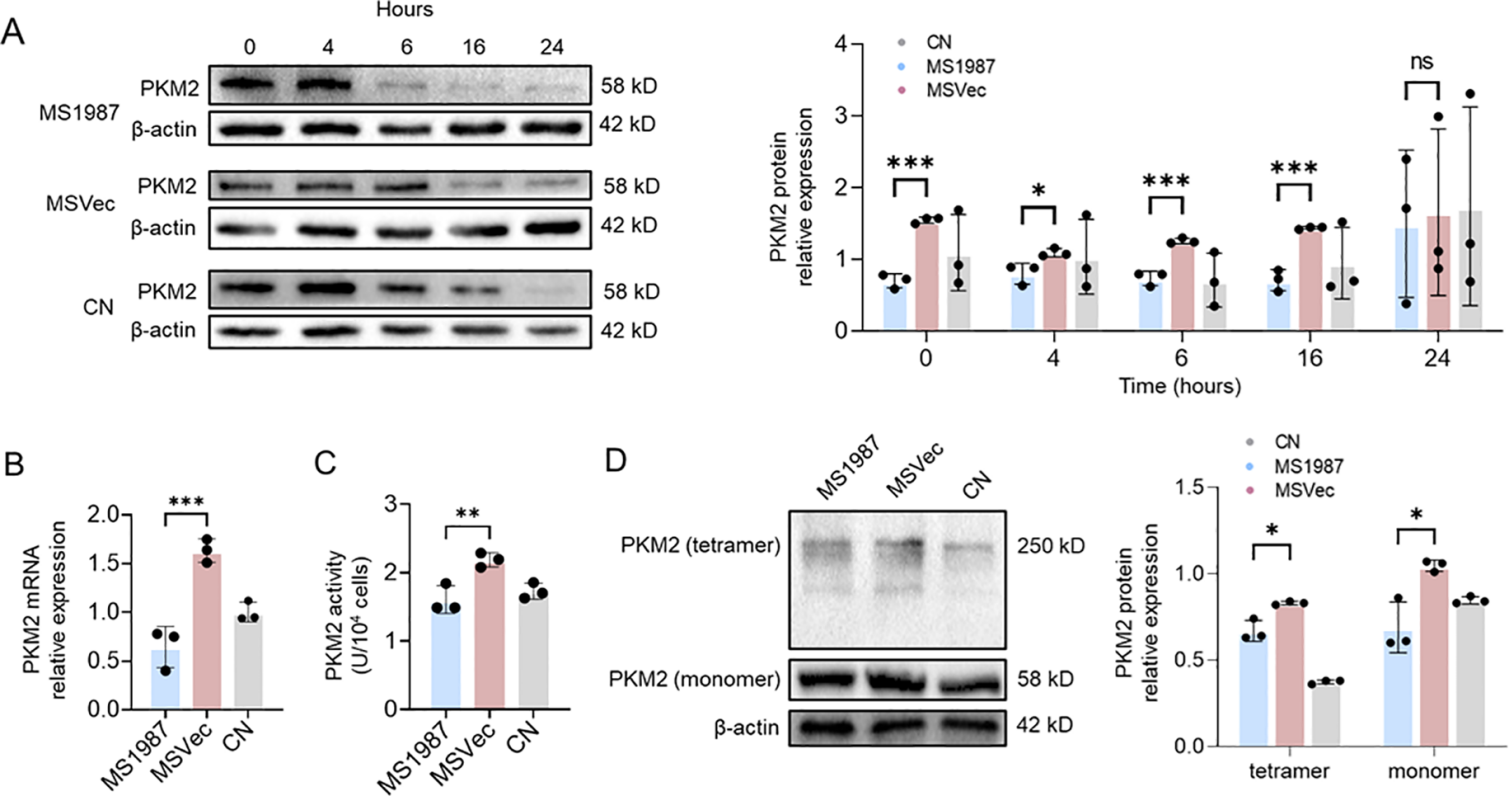
The expression, enzymatic activity and tetramerization of PKM2 were suppressed in Rv1987-induced M2 macrophages. The RAW264.7 cells were infected by MS1987 and MSVec for 3 hours and then collected at different time points. **(A)** Detection of the expression of PKM2 by Western blot at 0, 4, 6, 16, and 24 hours post-infection. **(B)** Detection of mRNA levels of PKM2 at 6 hours post-infection by RT-qPCR. **(C)** The enzyme activity of PKM2 at 6 hours post-infection. **(D)** Detection of the tetramer of PKM2 at 6 hours post-infection by DSS treatment followed by Western blot. The data are shown as the mean ± SD (n = 3), pooled from three independent experiments. Mean values were compared using one-way ANOVA analysis with *post-hoc* Tukey’s multiple comparisons test (*P < 0.05; **P < 0.01; ***P < 0.001; ns, no significant difference). *M. smegmatis* overexpressing Mtb Rv1987 protein; MSVec, *M. smegmatis* containing empty vector pVV2; CN, control group without infection.

As glycolysis and pyruvate metabolism were found to be significantly altered in Rv1987-induced M2-like macrophages, we focused on the effect of PKM2, the key enzyme involved in glycolysis and pyruvate metabolism. The expression of PKM2 in different time points after MS1987 and MSVec infection in RAW264.7 cells were detected firstly. The results showed that the expression of PKM2 was significantly impaired in MS1987 induced M2 macrophages at 4–16 hours post infection, especially at the time point of 6 hours (**Figure 2A**). RT-qPCR detection showed that the mRNA level of PKM2 was also decreased in MS1987 group than that in MSVec group (**Figure 2B**). The enzyme activity of PKM2 was further determined and the results showed that the PKM2 activity was obviously decreased in MS1987-infected macrophages compared to that in MSVec-infected cells (**Figure 2C**). As PKM2 have different confirmations and the tetramer confirmation contributes to the PKM2 enzyme activity, the tetramer level of PKM2 was detected by DSS cross-linking experiment. The results showed that tetramer confirmation of PKM2 was significantly decreased in MS1987-infected cells compared to MSVec-infected cells (**Figure 2D**), which further confirmed that PKM2 activity was suppressed in the M2 polarization induced by Mtb Rv1987 protein.

### The nuclear translocation of PKM2 and IL-1β production were impaired in Rv1987-induced M2 macrophages

**Figure 3:**
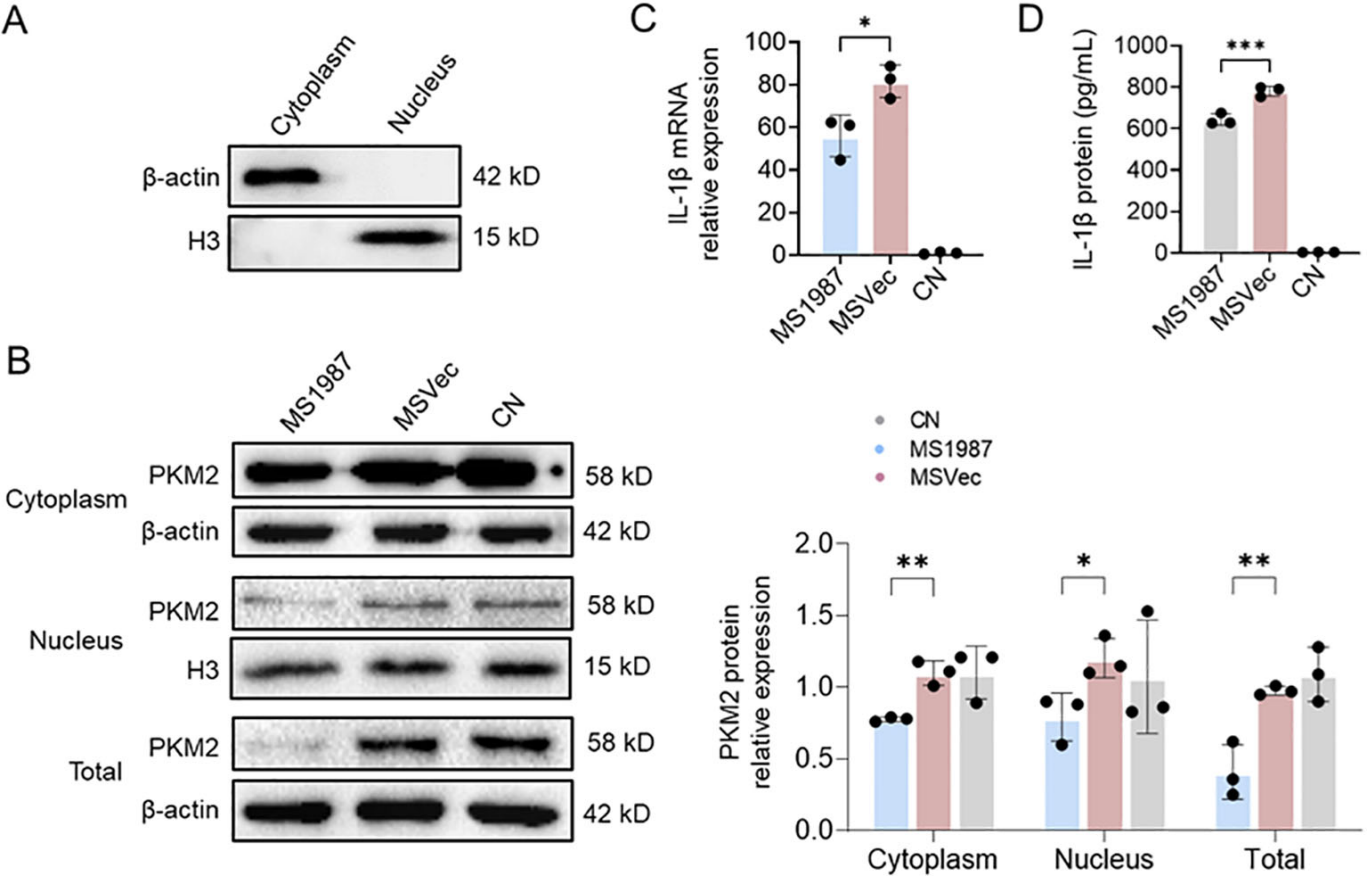
The nuclear translocation of PKM2 and the production of IL-1β were impaired in Rv1987-induced M2 macrophages. The RAW264.7 cells were infected by MS1987 and MSVec for 3 hours and the cell culture was collected at 6 hours post-infection. **(A)** Detection of β-actin and H3 in the isolated cytoplasm and nucleus fractions by Western blot. **(B)** Detection of the PKM2 level in cytoplasm and nucleus by Western blot. **(C, D)** Detection of intracellular IL-1β mRNA by RT-qPCR **(C)** and secretory IL-1β by ELISA **(D)**. The data are shown as the mean ± SD (n = 3), pooled from three independent experiments. Mean values were compared using one-way ANOVA analysis with *post-hoc* Tukey’s multiple comparisons test (*P < 0.05; **P < 0.01; ***P < 0.001). *M. smegmatis* overexpressing Mtb Rv1987 protein; MSVec, *M. smegmatis* containing empty vector pVV2; CN, control group without infection.

It is known that the nuclear translocation of PKM2 from cytoplasm to nucleus influences the inflammation response of macrophages. Therefore, the PKM2 levels in cytoplasm and nucleus of MS1987-infected macrophages were analyzed by isolating different cellular fractions. The results showed that cytoplasmic referent protein β-actin was only detected in cytoplasm fraction and nucleic referent protein H3 only present in nucleus fraction (**Figure 3A**), indicating the successful separation of cytoplasm and nucleus. On this basis, the PKM2 expression in cytoplasm and nucleus were analyzed. It was found that the PKM2 level was impaired in MS1987-infected macrophages compared to MSVec-infected cells, both in cytoplasm and nucleus, especially in the nucleus (**Figure 3B**). The production of pro-inflammation cytokine IL-1β was also lower in MS1987-infected macrophages than that in MSVec-infected cells both in mRNA (**Figure 3C**) and protein levels (**Figure 3D**). All these results suggested that the nucleus translocation of PKM2 was impaired in Rv1987-induced M2-like macrophages, which may subsequently inhibit the inflammation response of macrophages.

### PKM2 activator TEPP-46 reversed Rv1987-induced M2 polarization of macrophages

**Figure 4:**
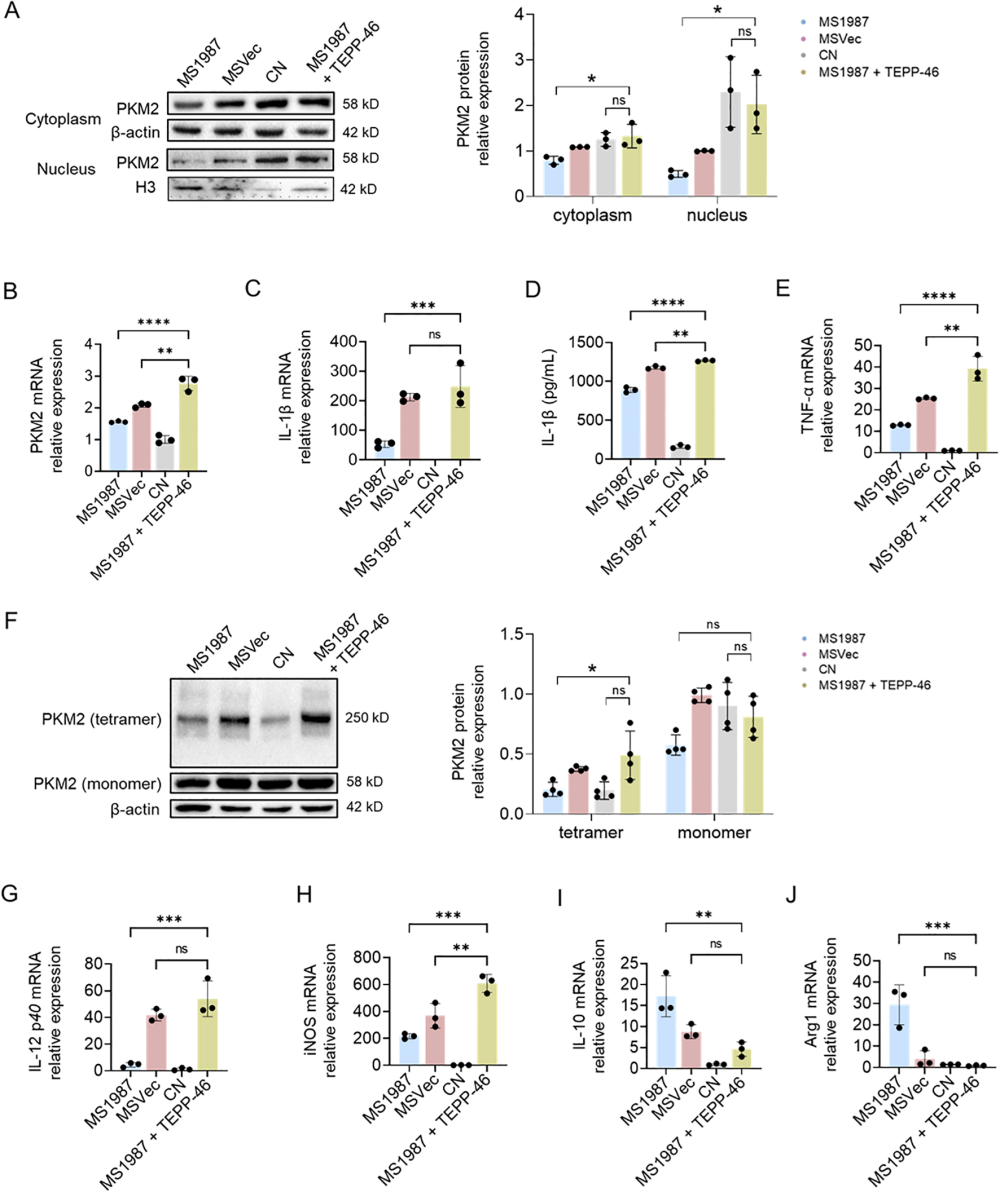
PKM2 activator TEPP-46 reversed Rv1987-induced M2 polarization of macrophages. RAW264.7 cells were infected with MS1987 or MSVec for 3 hours, and analyzed at 6 hours post-infection. In MS1987 + TEPP-46 group, RAW264.7 cells were pre-treated with 50 μM TEPP-46 followed by the infection with MS1987 bacteria. **(A)** The expression of PKM2 in the cytoplasm and nucleus. **(B)** The mRNA level of PKM2. **(C)** The mRNA level of IL-1β. **(D)** The protein level of IL-1β. **(E)** The mRNA level of TNF-α. **(F)** The tetramer of PKM2. **(G, H)** The mRNA level of IL-12 *p40***(G)**, iNOS **(H)**, IL-10 **(I)** and Arg1 **(J)**. The data are shown as the mean ± SD (n = 3), pooled from three independent experiments. Mean values were compared using one-way ANOVA analysis with *post-hoc* Tukey’s multiple comparisons test (*P < 0.05; **P < 0.01; ***P < 0.001; ****P < 0.0001; ns, no significant difference). *M. smegmatis* overexpressing Mtb Rv1987 protein; MSVec, *M. smegmatis* containing empty vector pVV2; CN, control group without infection.

To further confirm the effect of PKM2 on Rv1987-induced M2 polarization of macrophages, RAW267.4 cells were treated with TEPP-46 before infection. TEPP-46 is a classic PKM2 activator that can stabilize tetramerization of PKM2 in cytoplasm and limit inflammation of LPS-activated macrophages. It can also regulate NLRP3 inflammasome-mediated IL-1β secretion (Le et al., 2020). Different concentration of TEPP-46 was firstly used to evaluate its effect on PKM2 expression and it was found that PKM2 has the highest expression and enzymatic activity at the presence of 50 μM TEPP-46 (**Supplementary Figure 1**). A study also reported that 50 μM is the best concentration for activating PKM2 (Yi et al., 2021). Therefore, 50 μM TEPP-46 was used to treat RAW264.7 cells in the following experiments. The results showed that TEPP-46 significantly promoted the PKM2 expression both in cytoplasm and nucleus of MS1987-infected macrophages (**Figure 4A**). The total mRNA level of PKM2 was also increased (**Figure 4B**). The mRNA and protein levels of IL-1β were all elevated probably because more PKM2 translated into nucleus (**Figures 4C, D**). Another inflammation related cytokine TNF-α was also induced with TEPP-46 treatment (**Figure 4E**). DSS experiment showed that TEPP-46 enhanced the tetramerization of PKM2 in MS1987-infected macrophages (**Figure 4F**), indicating an enhanced enzyme activity of PKM2. The influence of TEPP-46 on Rv1987-induced macrophage polarization was then analyzed. The results showed that TEPP-46 treatment significantly promoted the production of M1 markers IL-12 *p40* and iNOS, meanwhile significantly inhibited the generation of M2 markers IL-10 and Arg1 (**Figures 4G**–**J**). All these results suggested that TEPP-46 reversed Rv1987-induced M2 polarization of macrophages and promoted macrophage inflammation by upregulation PKM2 expression and elevating PKM2 level in nucleus.

### PKM2 activator TEPP-46 inhibited mycobacterial growth *in vivo*

**Figure 5:**
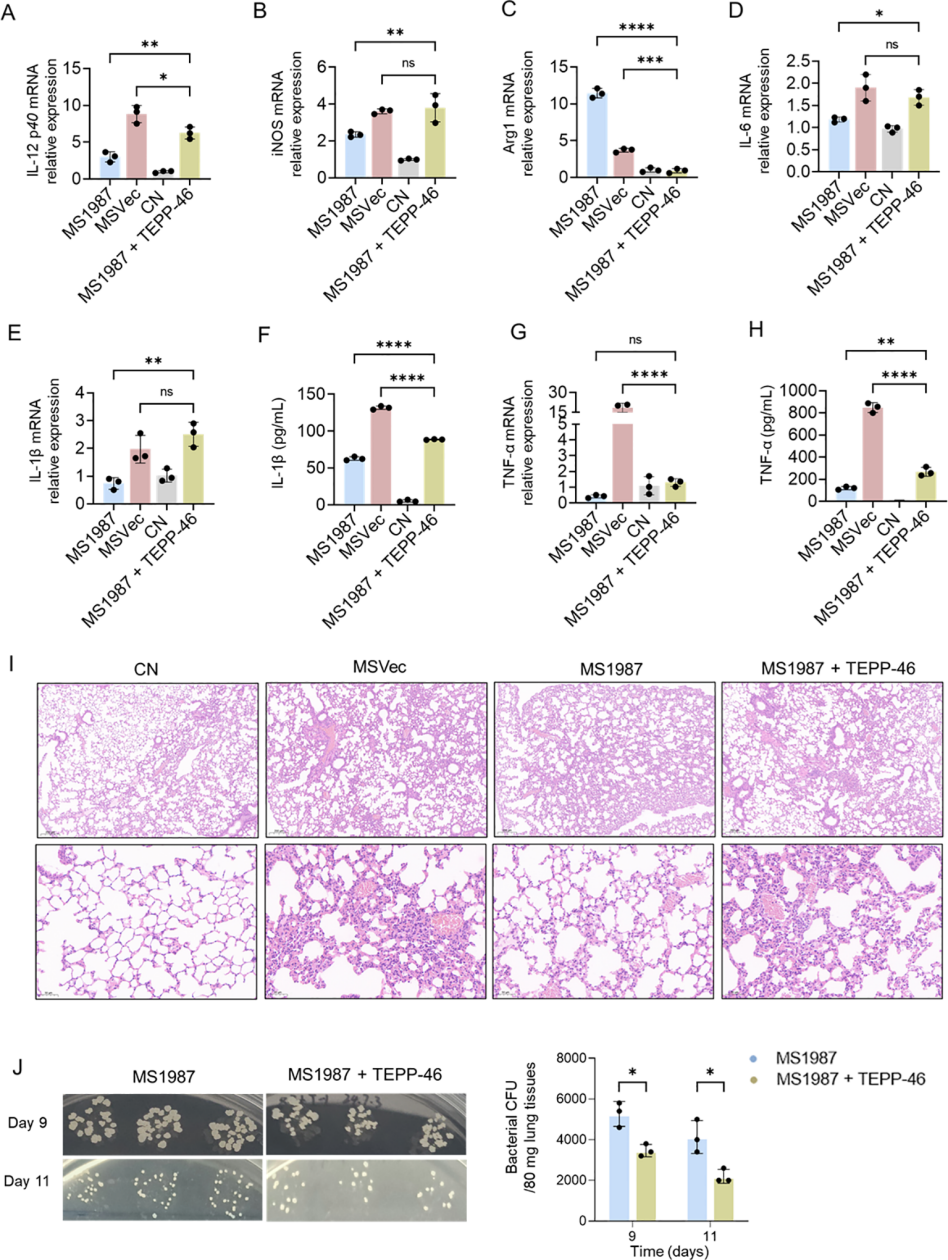
PKM2 activator TEPP-46 inhibited mycobacterial growth *in vivo*. C57BL/6J mice were infected with MS1987 or MSVec for 7 days at 5×10^9^ CFU/day by inhalation and analyzed at day 9 (for cytokines in lung tissues and serum, lung histopathology and bacterial CFU) and day 11 (for bacterial CFU) after the first day of infection. In MS1987 + TEPP-46 group, the mice were treated with 10 mg/kg TEPP-46 by intraperitoneal injection at 4 hours before infection with MS1987 bacteria. **(A–E)**, **(G)** Detection of the expression of IL-12, iNOS, Arg1, IL-6, IL-1β and TNF-α in mouse lung by RT-qPCR. **(F)**, **(H)** Detection of serum IL-1β and TNF-α by ELISA. **(I)** Lung histopathology by H&E staining. **(J)** Bacterial CFU in mouse lung. The data in **(A–H)** and **(J)** are shown as the mean ± SD (n = 3), pooled from three mice of each group. Mean values were compared using one-way ANOVA analysis with *post-hoc* Tukey’s multiple comparisons test (*P < 0.05; **P < 0.01; ***P < 0.001; ****P < 0.0001; ns, no significant difference). *M. smegmatis* overexpressing Mtb Rv1987 protein; MSVec, *M. smegmatis* containing empty vector pVV2; CN, control group without infection.

Our previous study showed that Rv1987 induced M2 polarization of alveolar macrophages (Sha et al., 2021) and enhanced mycobacterial growth in mouse lung (Sha et al., 2017). As TEPP-46 has been proved to be able to reverse macrophage M2 polarization by Rv1987 *in vitro*, we further detect whether TEPP-46 can inhibit mycobacterial growth *in vivo*. The mice were treated with TEPP-46 by intraperitoneal injection during MS1987 infection, and the results showed that at day 9 post-infection, compared with MS1987-infected mice without TEPP-46 treatment, IL-12 *p40* and iNOS expression were significantly increased and Arg1 production was obviously impaired in MS1987-infected mice with TEPP-46 treatment (**Figures 5A**–**C**). At day 11 post-infection, the changes in IL-12 *p40* and Arg1 were similar to those at day 9 post-infection, but iNOS decreased in TEPP-46 treatment group and had no significant difference from that in MS1987 group (**Supplementary Figure 2**). As for inflammatory cytokines, the IL-6 expression in MS1987-infected mouse lung tissues with TEPP-46 treatment was significantly higher than that without TEPP-46 treatment at day 9 post-infection (**Figure 5D**). IL-1β expression was increased both in lung tissues (**Figure 5E**) and serum (**Figure 5F**). The IL-1β level in the lung tissues of the mice infected and treated by MS1987 and TEPP-46 is even higher than that in MSVec group; however, in serum, it did not increase to the same extent, which probably because the lung tissue damage in the mice infected and treated by MS1987 and TEPP-46 was not as severe as that in MSVec group, which led to a tender increase of IL-1β in the serum. There is a trend toward increased TNF-α expression in lung tissues (**Figure 5G**), but a significant increase in serum (**Figure 5H**). Lung histopathology by H&E staining showed that compared with MSVec-infected mice, the lung tissues of MS1987-infected mice have a less inflammatory response after 7 days infection, which facilitated mycobacterial growth; however, with the treatment of TEPP-46, the mice perform a positive immune response with more lymphocytic infiltration (**Figure 5I**). The bacterial CFU results showed that the bacterial load in TEPP-46 treatment mice was significantly low than that in none TEPP-46 treatment mice, both at day 9 and day 11 after MS1987 infection (**Figure 5J**). These results suggested that TEPP-46 can inhibit mycobacterial growth *in vivo*, which was realized by promoting the expressions of M1 macrophage markers and stimulating the production of inflammatory cytokines.

## Discussion

In recent years, TB is found to be associated with metabolome variations in sputum (Beukes et al, 2023), urine (Izquierdo-Garcia et al., 2020; Pretorius and Luies, 2024) and blood (Albors-Vaquer et al., 2020; Collins et al., 2021), indicating that host metabolism plays an important role in TB development. Amino acids, carbohydrates and their metabolites are the most different compounds between TB positive and TB negative individuals. Some studies provided direct evidence that Mtb and its virulent factors can reprogram the metabolism and consequently regulate the differentiation and function of immune cells. For example, Mtb infection increases the uptake of low-density lipoprotein (LDL) and cholesterol by macrophages, and enhances *de novo* cholesterol synthesis in macrophages (Chen et al., 2024). Another research showed that the Mtb virulence lipid PDIM inhibits autophagy in mice, thus contributes to Mtb virulence and immune evasion (Mittal et al., 2024). Some studies also investigated the immune regulation functions of Mtb proteins, such as PPE31, which promote host cell death dependent on JNK signaling (Feng et al., 2021), and Rv2653, which stimulates host inflammation response by enhancing glycolysis in macrophages (Du et al., 2023). In our previous study, we found Mtb virulent factor Rv1987 protein induces M2 polarization through PI3K/Akt1/mTOR signal pathway (Sha et al., 2021), but the metabolic alternations and the function of key enzymes involved in this process, remains poorly understood.

PKM2, the key enzyme in glycolysis, has been reported to influence the polarization and inflammation of macrophages in various diseases. In ankylosing spondylitis, PKM2 and glucose transporter 1 (GLUT1) were observed to be upregulated in ankylosing spondylitis-derived monocytes and monocyte-derived macrophages, especially in M1 macrophages (Weng et al., 2023). In liver fibrosis, it is found that follistatin-like protein 1 (FSTL1) can bind to PKM2 and subsequently enhance PKM2-dependent glycolysis and increased M1 polarization (Rao et al., 2022). In acute lung injury, it was reported that SUMO-specific peptidase 3 (SENP3) facilitated M1 macrophage polarization via the hypoxia-inducible factor (HIF-1α)/PKM2 axis in lipopolysaccharide-induced acute lung injury (He et al., 2023). In acute kidney injury, researchers clarified that ubiquitin-specific protease 25 (USP25) targeted PKM2 and subsequently regulated aerobic glycolysis and lactate production during M1-like polarization (Yang et al., 2023). In recent years, PKM2 was also found to be secreted by exosomes, which perform different regulation functions on immune cells and caner tumorigenesis. It is reported that hypoxic exosomal PKM2 promote lung cancer progression by inducing M2 polarization of macrophages by activating AMPK pathway (Zhou et al., 2022). Recently, exosomal PKM2 was found to induce tumor-associated macrophages to M2 phenotype, which promoted the proliferation, migration, and invasion of gastric cancer cells (Yuan et al., 2025). These finding suggested the diversity and complexity of PKM2 existence form and function, and the effect of PKM2 in bacterial-induced M1/M2 polarized macrophages may be different. All these reports implied that PKM2 plays a crucial part in human spontaneous diseases by regulating the function of immune cells such as macrophages; however, our knowledge about the effect of PKM2 on bacteria-induced macrophages except for LPS is still limited.

Mechanically, PKM2 regulates the M1/M2 polarization and cytokine secretion of macrophages through its pyruvate kinase activity of tetramers as well as its protein kinase activity and transcriptional co-activator function of dimers (Li et al., 2025). Some factors, such as increased glucose uptake and glycolytic flux, promote the dimerization of PKM2 and enable its translocation to nucleus, where it directly interacts with HIF-1α to regulate expression of pro-glycolytic enzymes, or phosphorylates the transcription factor STAT3 and STAT1, thus boosting pro-inflammation factors IL-6 and IL-1β production (Corcoran and O’Neill, 2016). On the other hand, the tetramerization of PKM2 are retained in the cytosol and performed the pyruvate kinase activity, supporting the final step of glycolysis. Phosphorylation and deacetylation promote the conversion of PKM2 from tetramer to dimer, whereas lactylation inhibits this conversion (Rao et al., 2022; Das Gupta et al., 2020). In this study, IL-1β production was found to decrease in Rv1987-induced M2 macrophages. It was likely because that the expression and nucleus translocation of PKM2 were impaired. Certainly, it cannot be ruled out other mechanisms for PKM2 in regulating the IL-1β expression in Rv1987-induced M2 macrophages. It has been reported that PKM2 can also promote IL-1β production through inflammasome activation (Xie et al., 2016) and enhanced NF-κB signaling (Sun et al., 2025).

Several studies have proved that the PKM2 activity and structure can be regulated pharmacologically. It is reported that a kind of natural anti−inflammatory compound, celastrol, binds to Cys424 of PKM2, which inhibits the enzyme activity of PKM2; meanwhile, it binds to Cys106 of PKM2, which reduces the secretion of IL-1β (Luo et al., 2022). Another research found that ROS promotes glycolysis and facilitating macrophage M1 polarization through phosphorylating PKM2 by vital effector kinases in the DNA damage response (Li et al., 2024). These results indicated that both increasing enzyme activity and enhancing nuclear translocation of PKM2 contribute to M1 polarization and inflammation, which are consistent with our results. More importantly, an anti-tuberculosis study found that poxvirus and its core peptide IAMP29 can trigger metabolic reprogramming toward glycolysis and interacted with PKM2, thus activating the NLRP3 inflammasome and IL-1β production in human monocytes and murine macrophages and finally promoting an antimicrobial response to rapidly growing non-tuberculous mycobacteria (Roh et al., 2024). These findings strongly suggest the potential of PKM2 activation in Mtb controls.

In this study, we used TEPP-46 in the *in vitro* and *in vivo* experiments and found that it reversed the M2 phenotype of Rv1987-induced macrophages to M1 phenotype, increased inflammatory cytokines, and increased inflammatory markers. Noteworthily, our findings about the TEPP-46 action on macrophages are different from LPS-activated M1 macrophages. It is reported that Annexin A5 can shift LPS&IFN-γ-induced hepatic macrophage from M1 to M2 phenotype by promoting PKM2 tetramer formation and inhibiting phosphorylation of PKM2 (Xu et al., 2020). Similarly, activation of PKM2 by TEPP-46 in LPS-activated macrophages, can increases IL-10 production (Toller-Kawahisa et al., 2025). While, in our study, we found that activation of TEPP-46 in Rv1987-induced macrophages resulted in the reverse from M2 to M1 phenotype, with an increase of M1 macrophage markers and a decrease of M2 macrophage markers such as IL-10. Besides, in our study, TEPP-46 treatment led to the increased PKM2 expression and nuclear localization of PKM2, which differ from the existing literature. It is probably because LPS-activated macrophages and Rv1987-induced M2 macrophages are fundamentally different. In LPS-activated macrophage, PKM2 is highly expressed and already activated. Addition of TEPP-46 in these cells forces PKM2 into its tetrameric form and keep in cytoplasm, which relatively impairs the nuclear translocation of PKM2 and correspondingly reduces the inflammation and reprograms macrophages to an M2-like phenotype. This may be a kind of compensation response for extremely activated M1 macrophages, by which they can avoid excessive inflammation. However, in Rv1987-medicated M2 macrophages, PKM2 expression is relatively low. The stabilization of PKM2 tetramer by TEPP-46 enhanced the enzymatic activity of PKM2, which promoted glycolysis and inflammation rapidly. The increased metabolites such as succinate and the pro-inflammation cytokine such as TNF-a may influence the expression and the nucleus translocation of PKM2 indirectly. It has been reported that TNF-α induces PKM2 expression in HT-29 cells (Tang et al., 2015), while succinate promotes the succinylation of PKM2 at lysine 125 and promotes its transition from tetrameric to dimeric states as a result (Wang et al., 2025). Therefore, in Rv1987-induced M2 macrophages, TEPP-46 was observed to significantly increase PKM2 level not only in the cytoplasm but also in the nucleus, which may be the indirect effects of broader metabolic reprogramming induced by TEPP-46, rather than a direct action of TEPP-46. These findings remind us an alternative therapeutic strategy for PKM2 against Mtb compared to that against other diseases.

Additionally, it would be noticed that the *in vivo* effects of TEPP-46 in this study cannot be attributed exclusively to macrophages because TEPP-46 was administered systemically and it is not macrophage-specific. Other immune cells or systemic metabolic effects may also contribute to this process. It has been reported in recent years that except for macrophages, TEPP-46 can also regulate the function of CD4^+^T cells (Angiari et al., 2020), NK cells (Walls et al., 2020), as well as the lung epithelial cells (Chang et al., 2025). However, most of the research focus on the autoimmune disease models and the results showed that TEPP-46 inhibits over-inflammation or suppresses autoimmunity. These models obviously different from our mycobacteria-infection model, in which TEPP-46 acts as a “balancer” and plays a role in promoting inflammation and immune activation probably by metabolic reprogramming.

In recent years, host-directed therapies (HDTs) have emerged as a promising adjunct to conventional anti-TB drugs, aiming to modulate the host immune response to control Mtb infection and overcome drug resistance (Mehta et al., 2022; Costa et al., 2021; Kolloli and Subbian, 2017). As a key mediator of metabolic reprogramming and immune regulation, PKM2 exhibits remarkable synergy with current HDTs via complementary mechanisms. For example, chronic Mtb infection always drives macrophages toward an M2 phenotype, suppressing antimicrobial function to facilitate bacterial survival. Activation of PKM2 may reverse this metabolic skew, which complements immune modulatory HDTs such as IFN-γ therapy. PKM2 activation amplifies IFN-γ-induced STAT1 phosphorylation and antimicrobial gene expression, overcoming the limited downstream signaling of IFN-γ in chronic TB. Similarly, activation of PKM2 targeting will synergize with anti-inflammatory HDTs (e.g., interleukin-10 blockers or TNF-α inhibitors) by balancing immune activation. While these agents mitigate pathological inflammation, PKM2 modulation ensures that the host retains sufficient antimicrobial capacity to control Mtb, avoiding the risk of immunosuppression-induced bacterial reactivation.

Limitations. In this study, we used *M. smegmatis* as an experimental model (Sparks et al., 2023) to elucidate the effect of *M. tuberculosis* Rv1987 protein on macrophage immunometabolism. However, it is notified *M. smegmatis* cannot fully recapitulate the immune and metabolism induced by virulent *M. tuberculosis* own to its non-pathogenic characteristics.

## Conclusions

**Figure 6:**
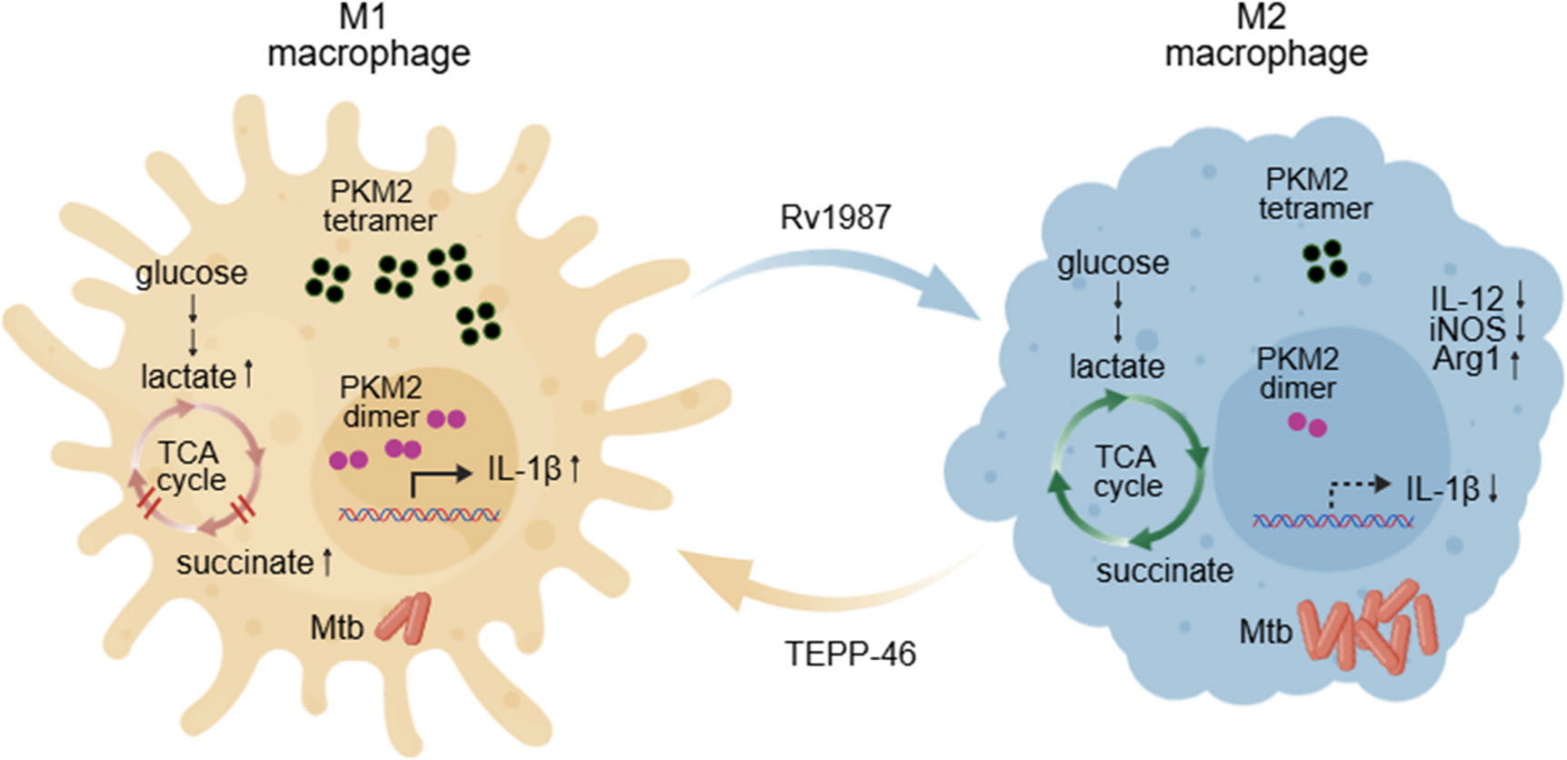
A schematic model summarizing the mechanism that Rv1987 modulates host metabolism and PKM2 function. Rv1987 protein decreases the accumulation of lactate, succinate and other intermediate products of TCA cycle in Rv1987-induced M2 macrophages, suggesting the enhanced flux through the TCA cycle. The expression and nucleus translocation of PKM2 are impaired in Rv1987-induced M2 macrophages, which may cause the decrease in IL-1β production and correspondingly provide a kindly environment for Mtb survival. TEPP-46 increases the expression and nucleus translocation of PKM2 in Rv1987-induced M2 macrophages, which reverses the M2 polarization and enhanced the inflammation of macrophages, and subsequently reduced the mycobacterial load in macrophages. This figure was created with Created with (accessed on January 20, 2026).

In summary, PKM2 is closely associated with the process of Mtb Rv1987-induced M2 polarization of macrophages. The expression, enzyme activity and nuclear translocation of PKM2 are impaired in these macrophages. Activation of PKM2 by TEPP-46 can re-polarize Rv1987-induced M2 macrophages to M1 phenotype and enhance the inflammation response of macrophages, and facilitates the host clearance of mycobacteria as a result (**Figure 6**). Our findings enhance the understanding about macrophage metabolism and its functions in mycobacterial infection, which provides a potential target and new strategy for anti-tuberculosis therapy from the host’s perspective.

## Data Availability

The original contributions presented in the study are included in the article/**Supplementary Material**. Further inquiries can be directed to the corresponding authors.
