# Supplementary figures and images for: Effect of PKM2 on M. tuberculosis Rv1987-induced macrophage M2 polarization
Source: Front Cell Infect Microbiol. 2026 Feb 13;16:1740892. doi: 10.3389/fcimb.2026.1740892 (PMC12945795; doi:10.3389/fcimb.2026.1740892)

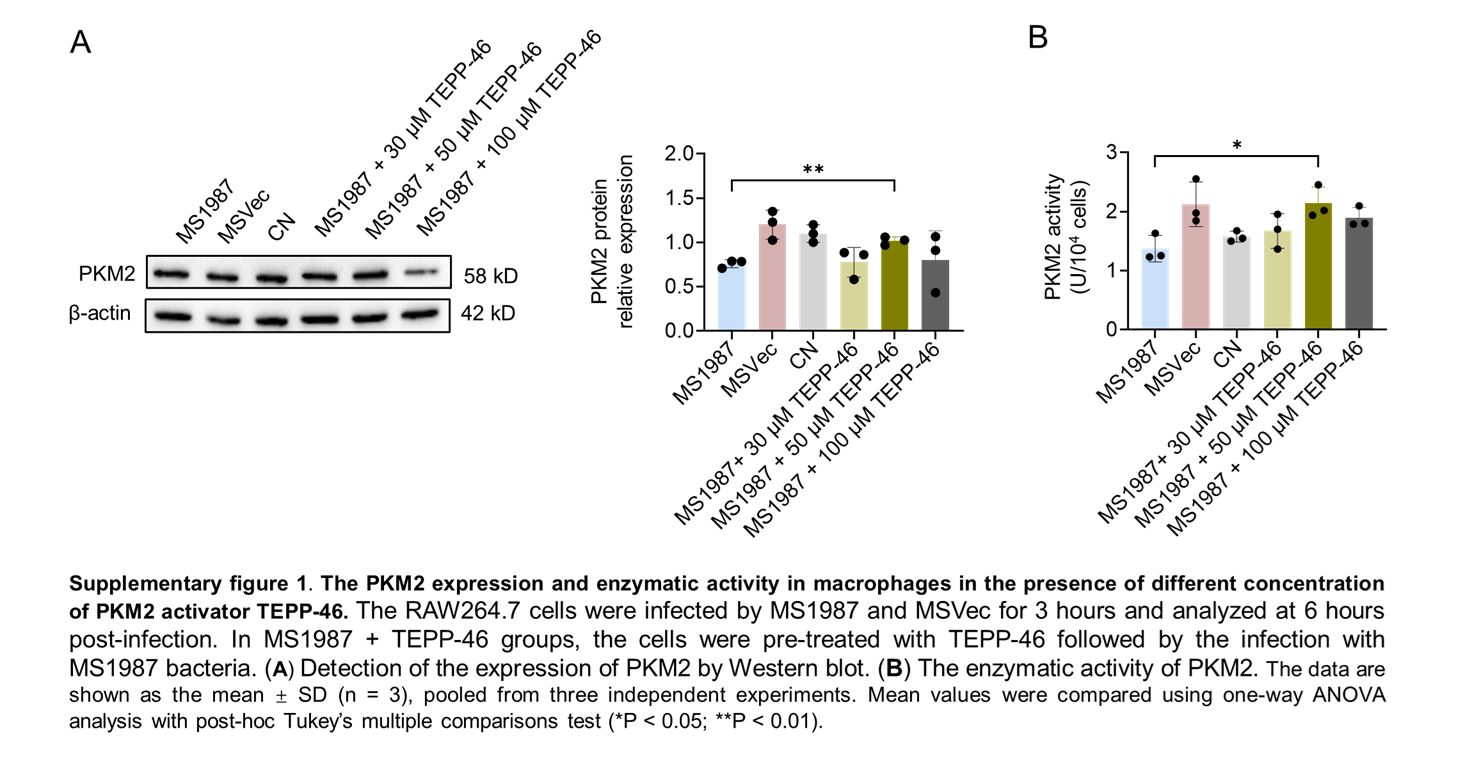

Supplement: Supplementary file 1 [file Image1.tif]

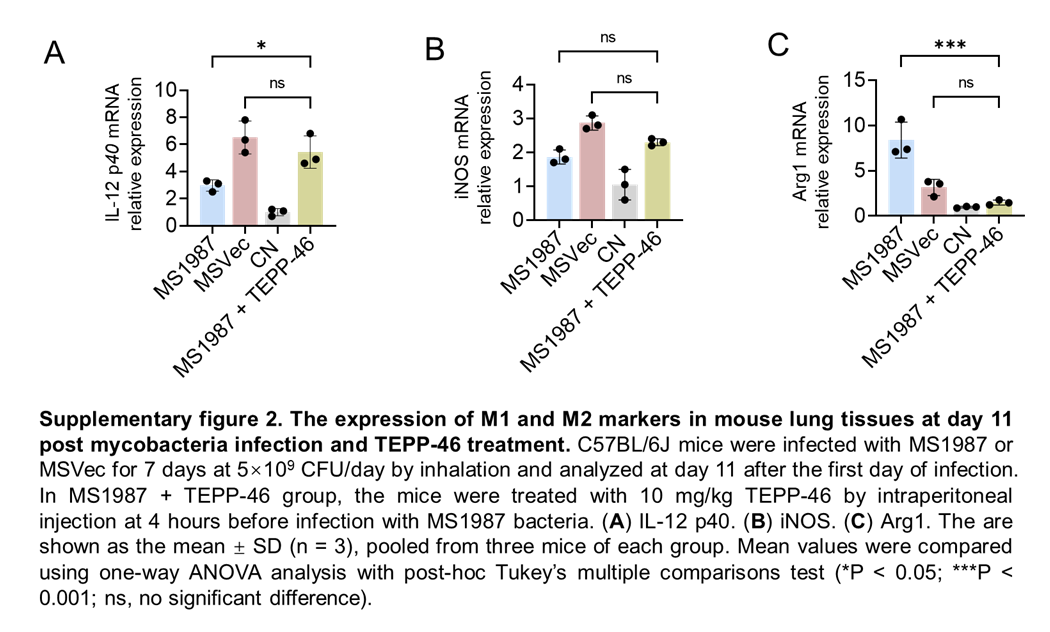

Supplement: Supplementary file 2 [file Image2.tif]
