# Supplementary material for: Effect of PKM2 on M. tuberculosis Rv1987-induced macrophage M2 polarization
Source: Front Cell Infect Microbiol. 2026 Feb 13;16:1740892. doi: 10.3389/fcimb.2026.1740892 (PMC12945795; doi:10.3389/fcimb.2026.1740892)
Supplement: Supplementary file 3 [file Table1.docx]

Supplementary table 1. Primers used for qPCR.

| Gene | Source | Primer sequence |
| --- | --- | --- |
| *IL-10* | mouse | 5’ AAGCCTTATCGGAAATGATCCA 3’  5’ GCTCCACTGCCTTGCTCTTATT 3’ |
| *IL-12 p40* | mouse | 5’ GAAGTTCAACATCAAGAGCAGTAG 3’  5’ GGACACTGAATACTTCTCAT 3’ |
| *iNOS* | mouse | 5’CCATCATGAACCCCAAGAGT 3’  5’ CTGGCCAGATGTTCCTCTAT 3’ |
| *Arg1* | mouse | 5’ AGTATGACGTGAGAGACCAC 3’ 5’ATTCTTCTGGACCTCTGCCA 3’ |
| *TNF-α* | mouse | 5’ CAGCCGATTTGCTATCTCATACC 3’  5’ GTACTTGGGCAGATTGACCTCAG 3’ |
| *IL-1β* | mouse | 5’ TGCCACCTTTTGACAGTGATGAG 3’  5’ TGATGTGCTGCTGCGAGATTT 3’ |
| *PKM2* | mouse | 5’ ccagtgatgg gatcatggtg 3’  5’ tgatcatgctc tccagcatc 3’ |
| *IL-6* | mouse | 5’ GAACAACGATGATGCACTTG 3’  5’ ATGTACTCCAGGTAGCTATG 3’ |
| *β-actin* | mouse | 5’ AGAGGGAAATCGTGCGTGAC 3’  5’ CAATAGTGATGACCTGGCCGT 3’ |
